# Editorial: Community series in post-translational modifications of proteins in cancer immunity and immunotherapy, volume III

**DOI:** 10.3389/fimmu.2024.1533926

**Published:** 2024-12-11

**Authors:** Xuejing Zhao, Naoe Taira Nihira, Xiangpeng Dai, Zichuan Liu

**Affiliations:** ^1^ School of Pharmaceutical Science and Technology, Faculty of Medicine, Tianjin University, Tianjin, China; ^2^ Tianjin University and Health-Biotech United Group Joint Laboratory of Innovative Drug Development and Translational Medicine, School of Pharmaceutical Science and Technology, Faculty of Medicine, Tianjin University, Tianjin, China; ^3^ Department of Translational Oncology, St. Marianna University Graduate School of Medicine, Kawasaki, Japan; ^4^ Key Laboratory of Organ Regeneration and Transplantation of Ministry of Education, First Hospital of Jilin University, Changchun, China; ^5^ National-Local Joint Engineering Laboratory of Animal Models for Human Disease, First Hospital of Jilin University, Changchun, China; ^6^ Frontiers Science Center for Synthetic Biology (Ministry of Education), Tianjin University, Tianjin, China

**Keywords:** protein post-translational modification (PTM), ubiquitination, E3 ligase, immunotherapy, cancer

Protein post-translational modification (PTM) plays a key role in the process of cancer initiation, development, progression and metastasis. PTMs are involved in signal transduction, gene expression regulation, and the formation of the tumor microenvironment by modulating the stability and function of critical proteins (Li et al.). Notably, the common types of PTMs include protein phosphorylation, ubiquitination, acetylation, methylation, glycosylation, sumoylation and palmitoylation (Ding et al.).

Ubiquitination is a prevalent PTMs that can regulate the fate of proteins by attaching ubiquitin molecules to target proteins via E3 ubiquitin ligases which determines the specificity of substrate proteins of ubiquitination. Therefore, the in-depth investigation of the function of E3 ligases and their interactions with other proteins is of great significance for revealing the complex protein regulatory network and the exploration of potential anti-tumor targets. As most papers listed in this Research Topic focused on PTMs regulated by E3 ligases. Therefore, we summarized the current pieces of evidences on PTMs mediated by E3 ligases in this Research Topic.

## E3 ubiquitin ligase as tumor suppressor

**Figure 1:**
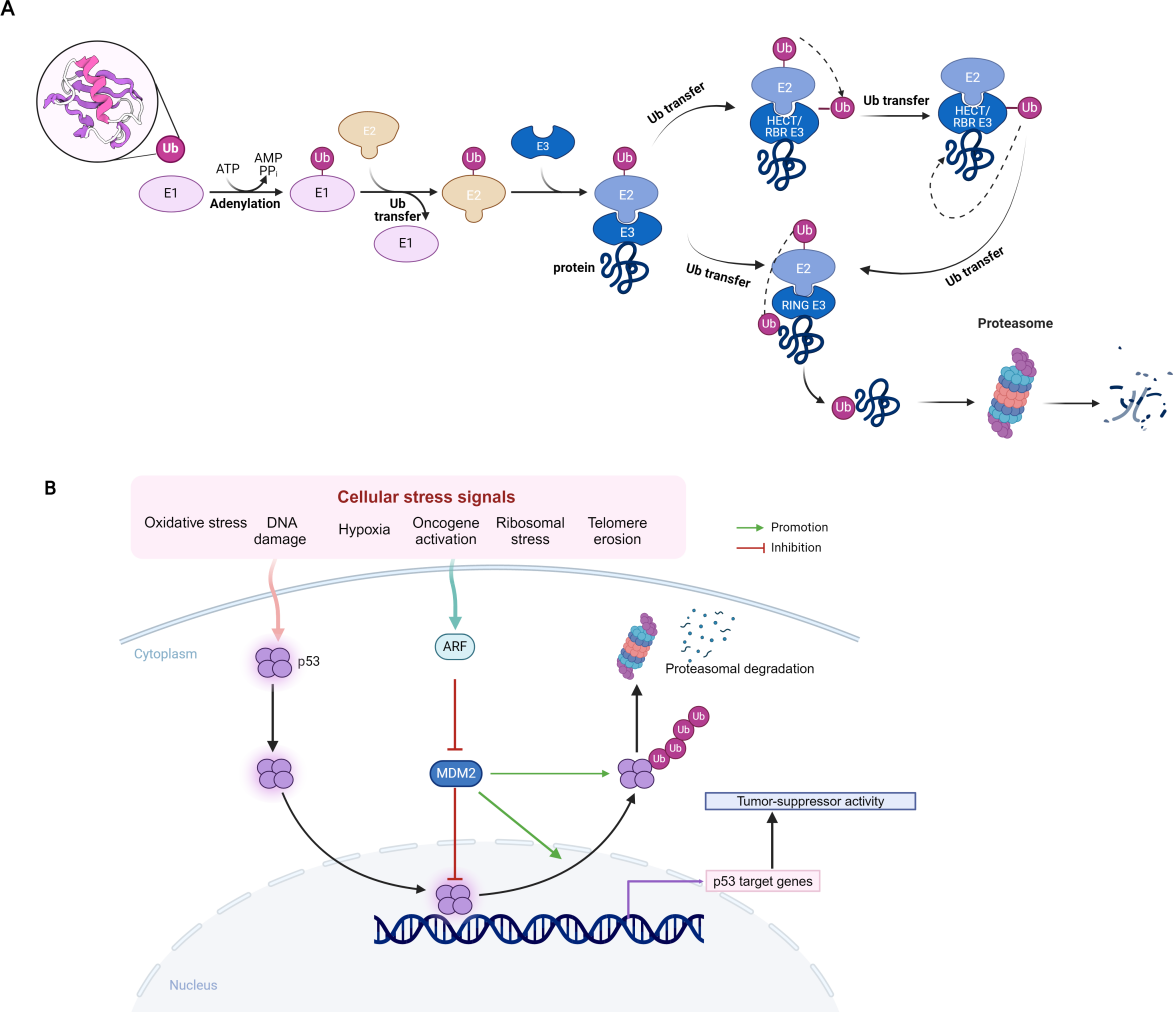
**(A)** Protein Ubiquitylation of E3 Ligases. **(B)** Ubiquitination of the MDM2-p53 pathway.

Currently, more than 800 proteins with E3 enzyme activity have been identified (1), and they are classified into several families according to their specific structural and functional features, including HECT, RING, RBR and U-box (2) (**Figure 1**). The E3 ligases can be tumor suppressors or oncogenes depending on their specific substrates. The E3 ligases with tumor suppressor role may target different substrate proteins in various cancers, ultimately leading to tumor suppression. In liver cancer, SOCS2 inhibits tumor progression by suppressing the JAK/STAT pathway and its overexpression reduces HCC cell invasion and migration. In HCC cells, Xu et al. showed SOCS2 induced ubiquitination of SLC7A11, and RIM46 regulated the ubiquitination process of GPX4, thereby inducing iron-dependent cell death and apoptosis, and ultimately inhibits cancer growth. Yin et al. indicated that another E3 ubiquitin ligase WDR37 may promote the degradation of TCP1 complex to inhibit tumor and improve immune cell infiltration, which can be used to accurately predict the prognosis of patients with pancreatic cancer.

## Histone ubiquitination

Interestingly, Duan et al. showed Histone H2A was the first identified ubiquitination substrate, with lysine 119 (H2AK119ub) serving as the single ubiquitination site, catalyzed by the polycomb repressive complex 1 (PRC1) E3 ligase Unconventional PTMs (3). Histone H2A ubiquitination (H2Aub) plays a key role in several biological processes, including gene transcription and DNA damage repair, thereby might affect the tumor development.

## Ubiquitination-like modifications

In recent years, the UFMylation, a protein posttranslational modification similar to ubiquitination, has been found to regulate various cellular processes. UFMylation involves the covalent attachment of ubiquitin-fold modifier 1 (UFM1) to target proteins (4). Ding et al. demonstrated that the UBA5 mediated UFMylation pathway influences various cellular processes including protein stability, cellular stress responses, and cellular signaling pathways. Dysregulation of UFMylation pathway is possible molecular mechanisms underlying some diseases, particular the neurodegenerative diseases and cancer. Moreover, the UFMylation of PD-1 facilitated tumor cell growth while the UFMylation of PD-L1, PLCA8 promoted cell apoptosis in breast cancer. Therefore, the in deep exploration of more substrates of UFMylation will shed light on the understanding of the critical role of UFMylation in cancer development or immunotherapy.

## T cell-related ubiquitination genes

T lymphocytes, integral to the adaptive immune system, wield pivotal influence in bolstering anti-tumor responses, and are strictly regulated by ubiquitination modification. Chen et al. developed a novel riskscore based on the close interaction between T cells and ubiquitination modification. In this study, they identified 5 core T cell marker genes, including UBE2E1, PSMD1, FBXL5, IVNS1ABP, and RNF10 via bioinformatic analysis. PSMD1 can affect HCC cell proliferation and apoptosis by influencing lipid droplet formation (5). FBXL5 can prevent iron overload and inhibit HCC occurrence (6), and inhibit HCC metastasis by suppressing snail expression levels. RNF10 has been confirmed to be a core gene predicting the prognosis of HCC patients, so the study focused on UBE2E1 and demonstrated its carcinogenic effect in HCC through a series of *in vitro* cell experiments.

## Conclusion

The E3 ligase and other proteins with PTM function reveal potential mechanisms for tumor therapy, and it can also accurately predict the prognosis of patients with a certain type of cancer, which make it to be used as a decision-making tool to guide therapy. Intensive study of these related modifications and their regulatory mechanisms can help develop new cancer therapeutic strategies.
